# Potential of root acid phosphatase activity to reduce phosphorus fertilization in maize cultivated in Brazil

**DOI:** 10.1371/journal.pone.0292542

**Published:** 2023-10-27

**Authors:** Lucas Lopes e Silva, João Antonio da Costa Andrade, Kátia Luciene Maltoni, Lucíola Santos Lannes

**Affiliations:** 1 Department of Biology and Animal Science, São Paulo State University, Ilha Solteira, São Paulo, Brazil; 2 Department of Plant Health, Rural Engineering and Soils, São Paulo University, Ilha Solteira, São Paulo, Brazil; Universidad Autónoma Agraria Antonio Narro, MEXICO

## Abstract

It is urgent to mitigate the environmental impacts resulting from agriculture, especially in highly biodiverse and threatened areas, as the Brazilian Cerrado. We aim to investigate whether root acid phosphatase activity is alternative plant strategies for nutrient acquisition in maize genotypes cultivated under fertilized and unfertilized conditions in Brazil, potentially contributing to reducing the use of phosphate fertilizers needed for production. Three experiments were performed: the first was conducted in a glasshouse, with 17 experimental maize inbred lines and two phosphorus (P) treatments; the second in the field, with three maize inbred lines and two treatments, one without fertilization and another with NPK fertilization; and the third was also carried out in the field, with 13 commercial hybrids, grown either under NK or under NPK treatment. Plant variables were measured and tested for the response to fertilization, differences amongst genotypes and response to root acid phosphatase activity. The activity of root acid phosphatase was modulated by the availability of P and nitrogen (N) in the soil and promoted grain filling of commercial hybrids in soils with low P availability. These results demonstrate that it is possible to select genotypes that are more adapted to low soil P availability aiming at organic production, or to use genotypes that have high phosphatase activity under P fertilization to reduce the amount of added P needed for maize production in Brazil.

## Introduction

Efforts towards sustainable agricultural production have received attention in recent years due to the urgent need to mitigate environmental degradation caused by agricultural practices [1–4]. Phosphorus (P) is limiting to plant growth in several agricultural systems, which is remediated by fertilizer applications, but phosphate fertilization when used in excess is harmful to the environment [5–9]. Reducing the use of phosphate fertilizers by exploiting organic P sources is an excellent alternative and one of the most promising means for more sustainable agricultural production, especially in Cerrado soils deficient in inorganic P but rich in organic P [10–12]. The Cerrado, as a rich reservoir of genetic resources, has wild plants with several adaptive strategies to overcome low P availability [13, 14], but cultivated plants also present a wide genotypic variation on strategies for P acquisition [15].

Plants can exploit various soil organic fractions through the exudation of acid phosphatases (PME), protons and organic acids, through greater root volume and investment in cluster roots and mycorrhizal association [16, 17]. PMEs are released by plants and microorganisms, having ideal activity in acidic conditions and increasing the general uptake of P by the plant. PME activity is responsible for active or appropriate mobilization of organic sources of P, cleaving phosphate from phosphomonoesters and phosphodiesters, increasing the uptake of the plant from P resources not readily available for uptake, making it possible to reduce the use of phosphate fertilizers on maize [18–20]. In fact, a considerable part of the assimilated P is produced from the mineralization of organic P through the PME [12].

Maize has high genetic variability and can be cultivated in different environmental conditions with specific and broad adaptations to the environment [21]. It has high productive potential and high demand for P due to its intense development and short cycle [22]. Studies point out that, amongst a wide range of PMEs codified by plants, purple acid phosphatases (PAPs) play a significant role in P foraging and recycling [23]. In maize, 33 genes related to PAPs were identified, showing that the expression of this character is quantitative and confirming that there are important functional variations within maize germplasm [24]. The authors also reported that the accumulation of the gene *ZmPAP26* was not different under high- and low-P availabilities, differently to 19 other PAP genes that had higher root acid phosphatase (rPME) activity under lower P availability. This suggests that PAP genes in maize have several functions in post-transcriptional regulation and possibly the functional divergence is higher than known so far.

Wang et al. [25] reported that maize had higher biomass and P concentration when grown with alfafa than in monocultures, both under P fertilization and control conditions, because alfafa produced more PME and anions in the mixture than in the monoculture, promoting an overall better soil exploitation. Sun et al. [26] studied PME and arbuscular mycorrhizae fungi (AMF) in maize grown alone and with alfafa and observed that maize PME was higher under lower soil P conditions and in the mixture, contributing to P uptake, upon which AMF had a limited effect. Another recent study showed that soil P, soil PME and maize productivity were not altered when phosphate fertilization was reduced in 20% added along with maize straw in relation to the conventional fertilization regime [20]. There were, therefore, several PME responses of maize to environmental and cultivation conditions even though these works still did not consider the existence of a wide genetic variation in maize for several characters, which is the basis for the development of genotypes well-adapted to varied environments and different cultivation regimes.

Based on the above assumptions, the main hypotheses of this research are that (1) rPME activity differs in various genotypes cultivated in the Cerrado, (2) rPME activity is modulated by inorganic P addition, with higher values found under natural cultivation than under traditional P addition, and (3) maize growth and productivity are affected by rPME. We aim at verifying the performance of various maize genotypes (experimental inbred lines and commercial hybrids) in relation to rPME under different soil inorganic P availabilities (S1 Fig). Specifically, we aim at investigating whether (1) different genotypes grown in the Cerrado have different rPMEs activities, (2) phenotypic plasticity for rPME in relation to fertilization occurs, (3) rPME affect growth and productivity under natural and P fertilized conditions.

## Materials and methods

### Glasshouse study

A glasshouse study was conducted from August to October 2018 in Ilha Solteira, São Paulo State, Brazil (20°25’04.77”S 51°20’30.65”W, 375 m elevation). We used 3.5 L pots filled with 3.2 kg dry soil of 2 mm-sieved Cerrado soil (dystrophic Red Latosol–Oxisol) [27]. From seeds, seventeen genotypes were cultivated under each of two treatments (control–only distilled water added, or P fertilization– 768 mg sodium phosphate (Na_2_HPO_4_), equivalent to 200 mg.P.kg^-1^ soil, as suggested by Novais et al. [28]), in three replicates, in a total of 102 pots. These inbred lines were chosen because they are experimental genotypes developed at São Paulo State University (UNESP–Campus of Ilha Solteira) and are potential candidates for a future breeding program (S1 Table).

In the 9-leaf stage of the plants, the pot was removed, the roots collected and washed first with tap water and then with distilled water. We then evaluated rPME activity, plant height, number of leaves, stem diameter, root water content, aerial dry biomass, root dry biomass and total dry biomass. The measurements were performed during this plant stage because it represents the end of the plant vegetative stage and the start of the most P-demanding stage of the crop.

Root PME activity was measured using 100 mg fresh roots in 5 ml p-NPP (*para*-nitrophenylphosphate). Root samples were taken to the laboratory for immediate measurements, and 3–5 analytical replicates were used per plant (p-NPP) bioassay [29]. Plant height and stem diameter were measured with a flexible ruler. Aerial dry biomass, root dry biomass, root plant water content and total dry biomass were measured after drying the material at 60°C for 72 h.

The chemical attributes of the soil used for the experiment are: 11 mg.dm^-3^ resin-P; 19 g.dm^-3^ organic matter; water-pH 5.0; K, Ca, Mg, H^+^, Al = 1.4; 11.0; 9.0 and 22.0 mmol_c_.dm^-3^, respectively; Cu, Fe, Mn, Zn = 1.6; 16.0; 20.0 and 0.7 mg.dm^-3^, respectively; 0.17 mg.dm^-3^ B, CEC = 43.4 0 mmol_c_.dm^-3^, 49% bases saturation and granulometry of 420, 50 and 530 g.kg^-1^ of sand, silt and clay respectively. Extractable P was measured colorimetrically after extraction with ion exchange resin and then washed with 0.8 M NH_4_Cl and 0.2 M HCl. N concentration was measured using the micro-Kjeldahl procedure. Extractable sulfur (S) was measured colorimetrically after extraction with activated charcoal and 0.01 M Ca(H_2_PO_4_). Soil pH was measured in a soil–water suspension (10 g dry soil in 50 ml deionized water) using a Metrohm Herisau pH meter with a Mettler Toledo electrode. Soil organic matter content was determined colorimetrically after extraction for 10 min with 0.667 M sodium dichromate and 5 M sulfuric acid. Soil extractable B was measured after extraction of 10 cm^−3^ dry soil with 20 ml barium chloride 6 mM solution by heating in a microwave at 490 W for 5 min. The B concentration was measured colorimetrically using the azomethine‐H method and adsorption at 420 nm on a spectrophotometer (Varian 50 Probe). Extractable Ca, Cu, Fe, Mg, Mn, K, and Zn concentrations were measured by means of atomic adsorption. Extractable Al was measured after extraction with 1 M KCl and titration with NaOH using the phenolphthalein method. All soil chemical characteristics were determined through standard methods at the UNESP Soil Laboratory according to Raij et al. [30], Lannes et al. [31] and Teixeira et al. [32]. At the end of the experiment, one soil sample per pot was collected for determinations of nutrients following the same methods.

### Field study 1

Field study 1 was performed from November 2018 to January 2019 in Selvíria, in the State of Mato Grosso do Sul, Brazil (20°20’50.65”S 51°24’06.32”W, 344 m elevation), located 11 km on the Northwestern of Glasshouse study area. Soils are classified as dystrophic Red Latosol–Oxisol [27]. Climate is characterized as Aw [33], tropical wet with a rainy season generally occurring from November to March and a pronounced dry season from April to October.

From seeds, we cultivated four genotypes under two fertilization treatments and three replications, in a total of 24 plants used for analysis. The genotypes were constituted by three inbred lines, L4, L8 and L12 (S1 Table)–which were selected because they had significantly lower rPME in the control than in P fertilized pots in the Glasshouse study, and a flint maize population (Pop), selected for low technology, genetically variable and equilibrated. The treatments used were control–only water added, or NPK fertilization– 20 kg.ha^-1^ (N): 51.6 kg.ha^-1^ (PO_4_): 33.2 kg.ha^-1^ (K). The plants were grown on lines with 3 meters length each with inter-row distance of 0.90 m (S2 Fig). This experiment was not randomized due to the aim of performing the cross-pollination manually, to obtain maize single-cross, where each genotype on its line was parallel with another line of an inbred line of interest for cross-pollination, with all possible combinations being carried out. The flint maize population was sown around the inbred lines and their harvest was random within each soil fertilization treatment (S2 Fig). To prevent water stress, all plots were irrigated two to three times a week according to the normal on-farm practice in this area, i.e., irrigation per demand following the evapotranspiration rate of the area according to our meteorological station (https://clima.feis.unesp.br/), which corresponds to a year average of 3 mm per day. In the 10-leaf stage of the plants we measured plant height and rPME using the abovementioned methods after removing parts of the roots using a shovel and washing first with tap water and then with distilled water.

At the end of the experiment, thirty top-20 cm soil samples were collected with a 5 cm diameter auger and combined in a composite sample. Samples were air dried, sieved and sent to the UNESP Soil Laboratory for determination of chemical attributes as previously described.

### Field study 2

A second field study was conducted from April to September 2019 in an area located close to where Field study 1 took place. We used 13 commercial hybrids (S2 Table), widely used in the region [34–44], with cycle is semi-early, early or super-early, single-cross and double-cross. In three randomized blocks and two treatments: Control–NK addition (20 kg.ha^-1^ (N): 0 (PO_4_): 33.2 kg.ha^-1^ (K)), or NPK fertilization (20 kg.ha^-1^ (N): 51.6 kg.ha^-1^ (PO_4_): 33.2 kg.ha^-1^ (K)). The plants were grown on 6 lines with 5 meters length each with inter-row distance of 0.45 m, in a total density equivalent to 60,000 plants per hectare (S3 Fig). To prevent water stress, all plots were irrigated two to three times a week according to the normal on-farm practice in this area. In the 10-leaf stage, using one random plant from each block (total of 78 plants) was used for measured rPME, number of leaves and root plant water content, and at the beginning of the reproductive stage, the plant height and ear height were measured using five random plants from each block. Ear height refers to height of insertion of the first ear and plant height is the height of the last fully open leaf. For the destructive methods, the whole field plant was gently removed from the soil using a shovel, the roots were washed first with tap water and then with distilled water. After harvest, we measured the weight of 100 randomly selected grains because this variable is less susceptible than grain weight per plant to other influences of P deprivation as pollination and diseases.

At the end of the experiment, thirty top-20 cm soil samples were collected with a 5 cm diameter auger and combined in a composite sample. Samples were air dried, sieved and sent to the UNESP Soil Laboratory under the current methods mentioned in the previous sections.

### Data analyses

The effect of fertilization upon measured variables was analysed through Student t tests and the differences amongst genotypes were assessed through ANOVA followed by Tukey test using IBM SPSS Statistics 20. Cohen’s d effect sizes [45] were calculated as standardized differences between means of total biomass, aerial biomass, root biomass, stem biomass, number of leaves, height and root phosphatase activity between P fertilized and control plots. Linear regression analyses were employed to assess the effects of rPME on growth and productivity parameters. Data were log transformed when necessary to reach normal distribution and homoscedasticity.

## Results

### Glasshouse study–Inbred lines in mesocosms

Fertilized pots had significantly higher available P concentration than unfertilized pots (respectively 71.3±32.5 and 11.7±0.58 mg.dm^-3^, t = 10.10, P = 0.034, more about soil characteristics in S3 Table), confirming that the fertilization treatment was effective. Phosphorus fertilization did not affect other soil variables, with exception to iron, whose concentrations also increased in the soil under P fertilization (Fertilized: 34.0±5.6 and Control: 21.3±2.3 mg.dm^-3^, t = 13.2, P = 0.022).

**Fig 1:**
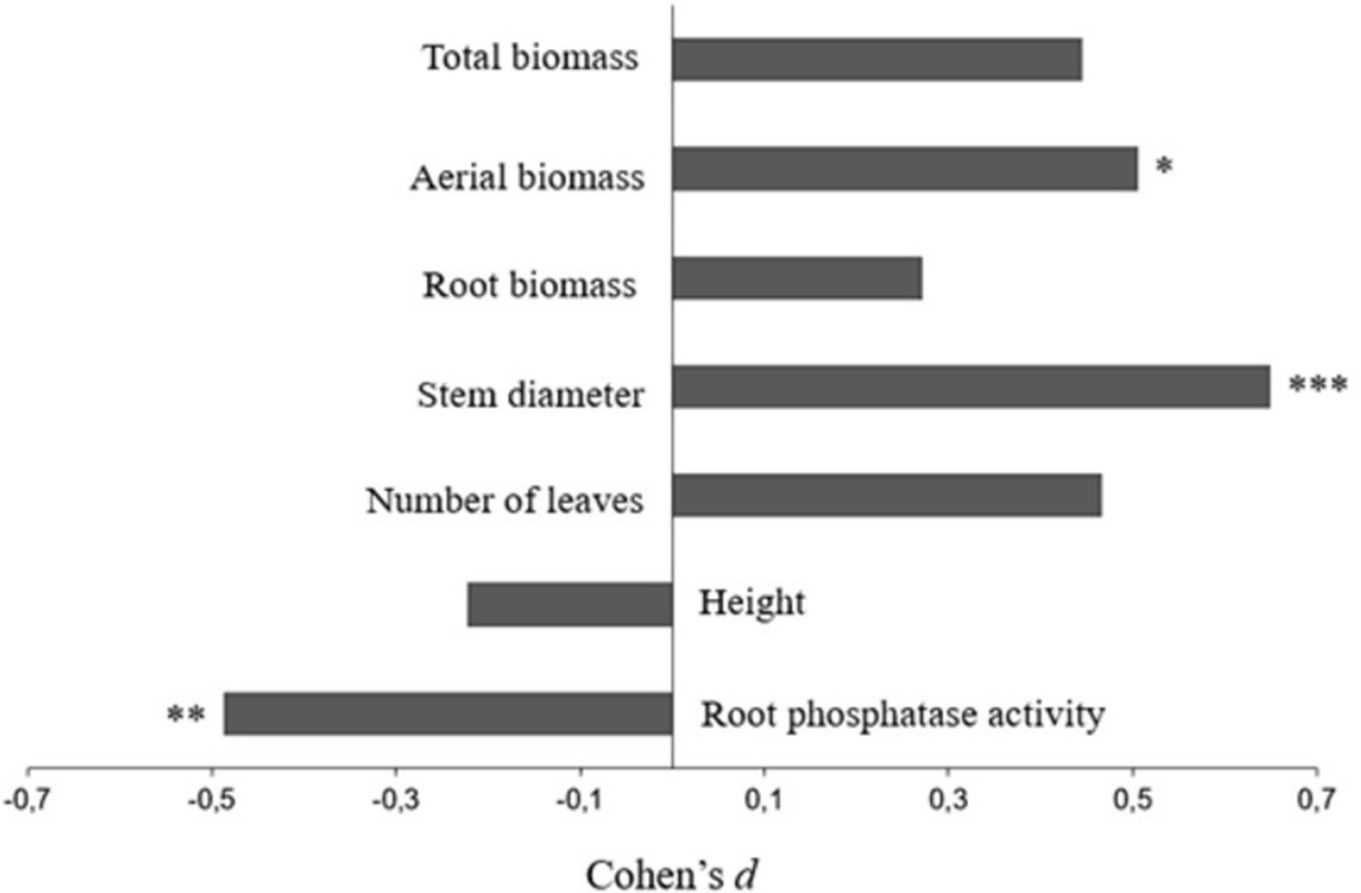
The effect sizes of P fertilization on measured variables in 17 inbred lines from the glasshouse study. Effect sizes of P fertilization (200 mg.P.kg^-1^ soil) on measured variables in the 9-leaf stage of maize (*Zea mays*). Positive values show that concentrations in P fertilized plots were higher than in the Control plots. Asterisks indicate significant differences (Student’s t test, * P<0.05, ** P<0.01, *** P<0.001) between P fertilized and Control plots. For full statistical results, see S4 Table.

Root PME activity significantly decreased under P fertilization when all inbred lines were analysed together (Fig 1). Oppositely, P fertilization had a positive influence of stem diameter and aerial dry biomass. Plant height, number of leaves, total and root dry biomass were not affected by P addition (Fig 1).

Six studied inbred lines (L4, L8, L9, L10, L12 e L14) had higher rPME activity in the control than in the fertilized pots, whereas the other eleven inbred lines (L1, L2, L3, L5, L6, L7, L11, L13, L15, L16 e L17) were not affected by P fertilization (Table 1). Although rPME generally decreased under P fertilization (Fig 1 and Table 1), the inbred lines showed highest differences amongst themselves under P fertilization, as observed in L3 (314±124 μmol pNPP g-root^-1^.h^-1^) and L4 (303±34 μmol pNPP g-root^-1^.h^-1^), which were significantly lower than L1 (779±462 μmol pNPP g-root^-1^.h^-1^), L9 (774±9 μmol pNPP g-root^-1^.h^-1^) and L11 (772±305 μmol pNPP g-root^-1^.h^-1^) (Table 1).

**Table 1:** The P fertilization effect on rPME in 17 inbred lines and in the Flint maize population (*Zea mays*) in the glasshouse study and in the field study 1.

Inbred lines	Glasshouse study					Field study 1		
	Control		P Fertilization		*Sig*	Control	NPK Fertilization	*Sig*
L1	552 (163)	a	779 (462)	bc	0.518	.	.	**.**
L2	526 (108)	a	391 (120)	abc	0.200	**.**	**.**	**.**
L3	448 (112)	a	314 (124)	a	0.248	**.**	**.**	**.**
L4	494 (109)	a	303 (34)	a	0.030	1754 (368)	1386 (359)	0.148
L5	504 (87)	a	452 (129)	abc	0.555	**.**	**.**	**.**
L6	531 (132)	a	649 (233)	abc	0.494	**.**	**.**	**.**
L7	491 (51)	a	421 (153)	abc	0.404	**.**	**.**	**.**
L8	691 (114)	a	392 (39)	abc	0.007	1173 (260)	1385 (916)	0.720
L9	933 (45)	a	774 (9)	c	0.003	**.**	**.**	**.**
L10	712 (12)	a	506 (52)	abc	0.005	**.**	**.**	**.**
L11	861 (747)	a	772 (305)	bc	0.873	**.**	**.**	**.**
L12	712 (129)	a	436 (96)	abc	0.044	2649 (1351)	1351 (208)	0.175
L13	469 (26)	a	432 (95)	abc	0.510	**.**	**.**	**.**
L14	617 (109)	a	356 (40)	ab	0.010	**.**	**.**	**.**
L15	637 (220)	a	527 (17)	abc	0.461	**.**	**.**	**.**
L16	601 (139)	a	607 (138)	abc	0.973	**.**	**.**	**.**
L17	594 (233)	a	478 (67)	abc	0.595	**.**	**.**	**.**
Flint population	**.**	**.**	**.**		**.**	1994 (536)	1637 (652)	0.198
Total	610 (224)	**.**	505 (207)		0.003	1913 (720)	1500 (569)	0.046
P and F (ANOVA)	P = 0.079		P<0.001			P = 0.069	P = 0.801	
	F = 1.790		F = 4.051			F = 2.831	F = 0.333	

Mean values, standard deviations (in parentheses) and significance values of the P fertilization effect (200 mg.P.kg^-1^ soil) on rPME (μmol pNPP g-root^-1^.h^-1^) were tested by means of Student t tests and the differences amongst the inbred lines were tested through ANOVA followed by Tukey tests. Different letters indicate significant differences amongst the inbred lines within treatments. (P<0.05), N = 3.

### Field study 1 –Inbred lines in the field

**Fig 2:**
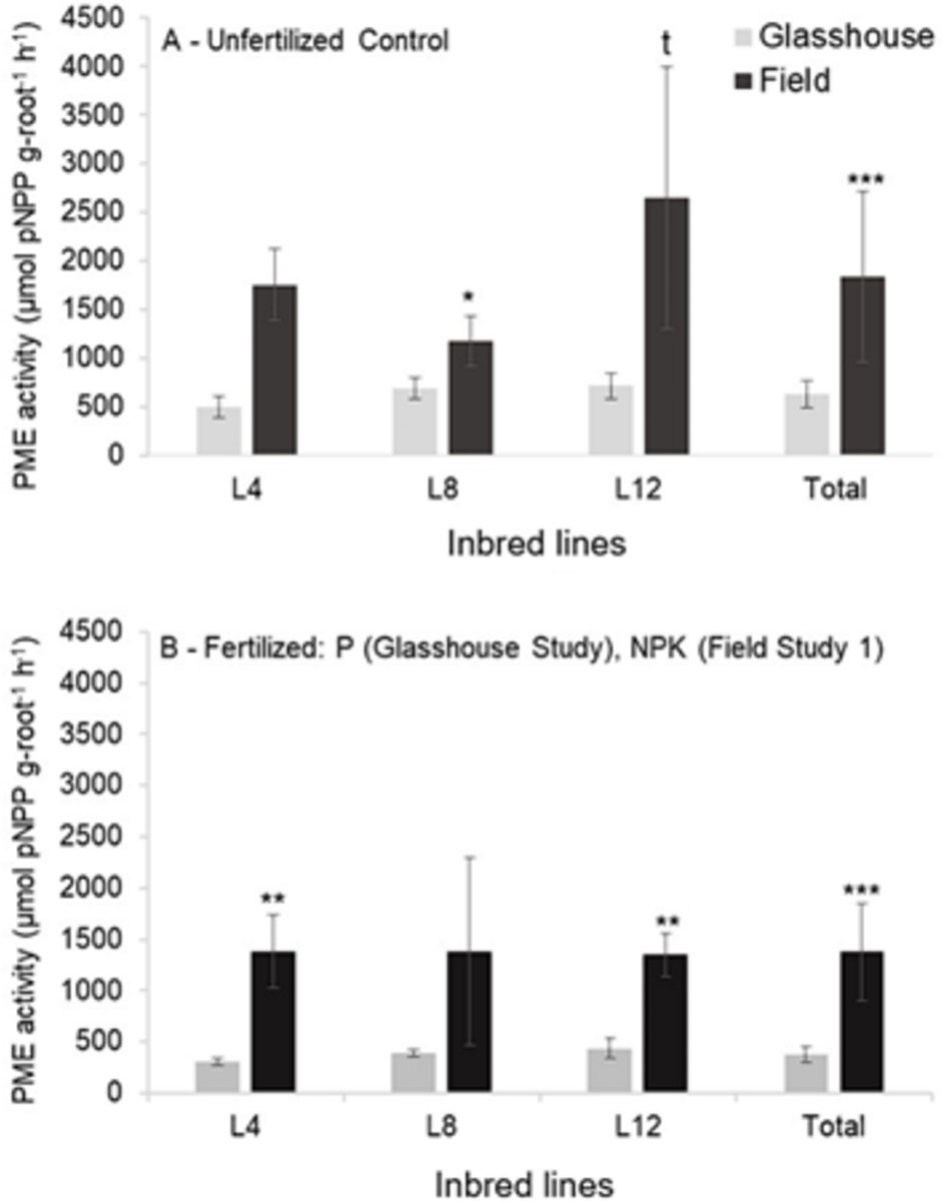
Root acid phosphastase activity of three individual inbred lines L4, L8 and L12, analysed together in the glasshouse study and in the field study 1 in the unfertilized controls and in the fertilized treatments. Means, standard deviations and levels of significance resulting from Student t tests are shown. t 0.10>P>0.05, * P<0.05, ** P<0.01, *** P<0.001.

The overall average rPME of the inbred lines cultivated in the field was higher in the controls that under NPK fertilization, but no inbred lines individually have shown such response (Table 1). Root PME activity was generally higher in the field than in the glasshouse study (Fig 2).

### Field study 2 –Commercial hybrids in the field

There was generally no effect of fertilization on rPME for the studied hybrids, with exception to hybrid 11, which had higher rPME in the fertilized than in control plants; no differences in rPME were observed amongst the hybrids (Table 2).

**Table 2:** The effects of NK addition or NPK fertilization on rPME and weight of 100 grains of 13 commercial hybrids of maize cultivated in the field study 2.

Hybrid	Root PME activity		Weight of 100 grains	
	Control	P fertilized	Control	P fertilized
H1	1012 (341)	885 (138)	35.7 (2.8) bc	40.8 (1.1)* c
H2	1168 (430)	1356 (548)	35.4 (0.6) bc	36.3 (2.0) abc
H3	835 (194)	641 (77)	37.9 (5.9) c	40.7 (1.4) c
H4	840 (435)	661 (217)	31.4 (3.4) abc	30.9 (2.2) ab
H5	1067 (244)	929 (374)	35.2 (7.2) bc	37.4 (1.6) bc
H6	799 (310)	885 (60)	31.9 (0.3) abc	30.5 (6.6) ab
H7	1011 (371)	842 (69)	34.1 (2.8) abc	38.0 (4.0) bc
H8	774 (290)	1199 (429)	26.9 (1.8) ab	33.3 (4.4) abc
H9	782 (620)	1090 (542)	28.6 (1.3) abc	32.2 (1.9)* abc
H10	575 (85)	715 (213)	30.4 (2.1) abc	29.0 (0.4) ab
H11	727 (90)	947 (66)*	24.9 (1.8) a	27.3 (3.3) a
H12	627 (284)	1150 (368)	30.6 (1.1) abc	29.8 (1.4) ab
H13	956 (190)	1150 (87)	29.5 (2.2) abc	31.8 (4.8) abc
Total	860 (313)	930 (330)	31.7 (4.5)	33.7 (5.1)
P and	P = 0.756	P = 0.157	P<0.001	P<0.001
F (ANOVA)	F = 0.679	F = 1.588	F = 4.19	F = 5.87

Mean values and standard deviations of rPME (μmol pNPP g-root^-1^.h^-1^) and weight of 100 grains (g) under two treatments: Control–NK addition (20 kg.ha^-1^ (N): 0 (PO_4_): 33.2 kg.ha^-1^ (K)), or NPK fertilization (20 kg.ha^-1^ (N): 51.6 kg.ha^-1^ (PO_4_): 33.2 kg.ha^-1^ (K)). The effects of fertilization upon measured variables were tested by means of Student t tests (N = 3) and indicated by asterisks when significant (* P<0.05, ** P<0.01). Differences among hybrids were analysed through ANOVA followed by Tukey test (N = 3) and different letters indicate differences within treatments (P<0.05).

The hybrids 1 and 9 had higher weight of 100 grains in the fertilized than in the control plants (Table 2). For plant height, the hybrids 1, 2, 3, 8, 9, 12 and the general mean were higher in the control than in fertilized plants. The ear height of hybrid 2 was higher in the control, and the opposite was observed for the hybrids 4 and 10 (S5 Table). The weight of 100 grains and the ear height were different amongst the hybrids in both treatments, and plant height only differed in the control plots (Table 2 and S5 Table).

**Fig 3:**
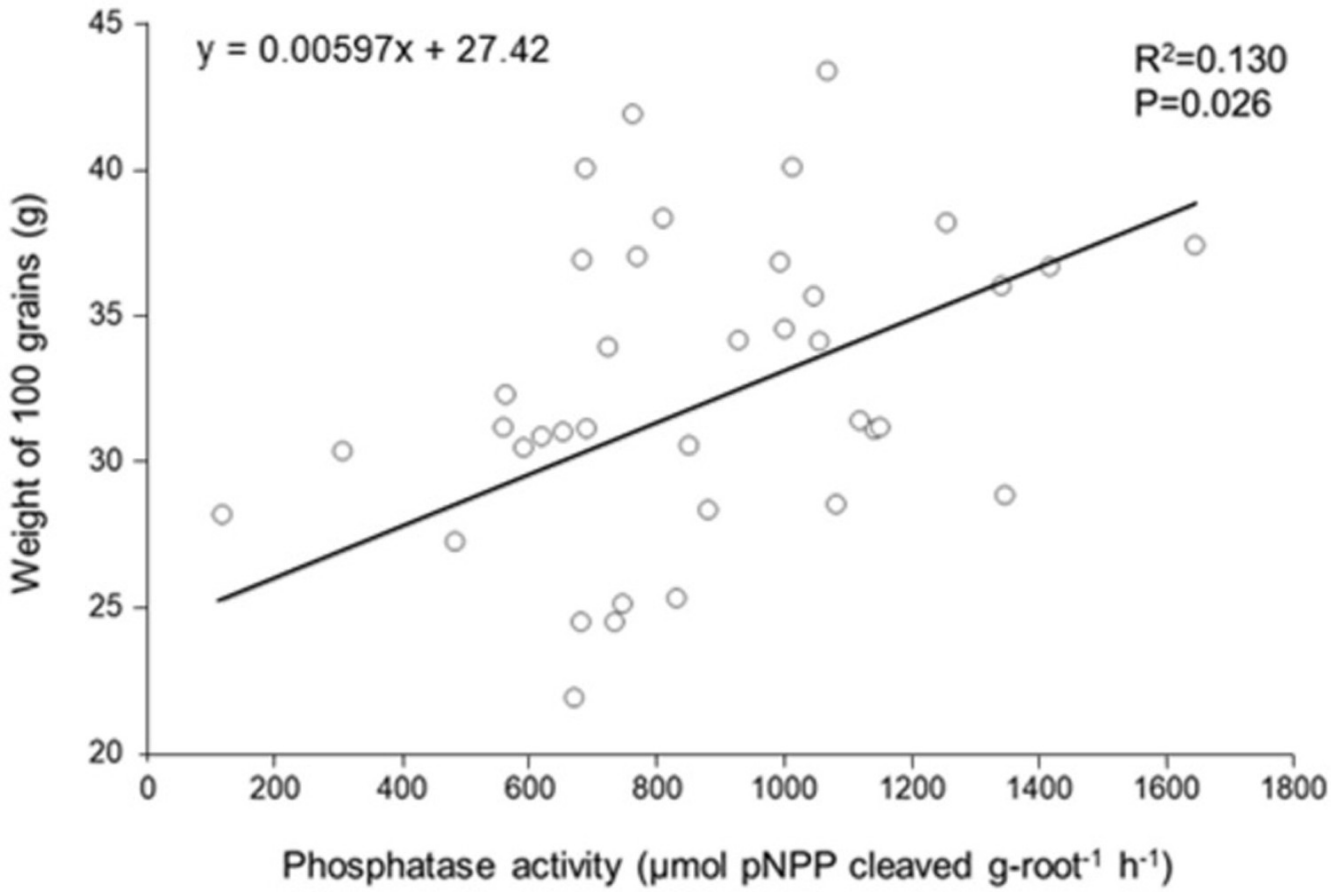
Effect of rPME on weight of 100 grains in P-unfertilized control plots in 13 commercial hybrids of maize (*Zea mays*) cultivated in the field (N = 39).

Root PME activity positively influenced the weight of 100 grains of the hybrids in the control plots (Fig 3), but it did not influence other variables (S6 Table).

### Soil characteristics

Soil P concentrations were not significantly different amongst the three experiments (Table 3). Nitrogen, copper and manganese concentrations were different in all experiments. Organic matter and potassium concentrations were higher in the field studies than in the glasshouse study, and the opposite was observed for pH. Calcium and magnesium concentrations were higher in the Field study 2 than in the other experiments, and zinc concentration was lower in the Glasshouse study that in the Field study 2 (Table 3).

**Table 3:** Soil characteristics in the three experiments performed.

	Glasshouse study	Field study 1	Field study 2	F	P
N (g.kg^-1^)	0.93 (0.07) a	1.39 (0.09) b	1.70 (0.08) c	117.55	<0.001
P—resin (mg.dm^-3^)	41.5 (38.6)	40.0 (13.5)	36.3 (7.3)	0.03	0.970
MO (g.dm^-3^)	19.3 (1.03) a	24.7 (1.15) b	26.3 (2.08) b	31.93	<0.001
pH (CaCl_2_)	5.03 (0.1) b	4.63 (0.15) a	4.73 (0.12) a	13.59	<0.001
K (mmol_c_.dm^-3^)	0.78 (0.08) a	5.06 (0.67) b	4.13 (0.71) b	105.39	<0.001
Ca (mmol_c_.dm^-3^)	12.3 (0.82) a	10.0 (1.73) a	16.3 (1.15) b	23.34	<0.001
Mg (mmol_c_.dm^-3^)	10.0 (0.63) a	9.7 (0.58) a	12.7 (1.53) b	10.79	<0.001
Al (mmol_c_.dm^-3^)	1.67 (0.82)	3.33 (1.53)	2.33 (0.58)	2.90	0.110
B (mg.dm^-3^)	0.15 (0.05)	0.19 (0.03)	0.17 (0.04)	0.80	0.480
Cu (mg.dm^-3^)	20.8 (1.3) a	52.7 (1.5) b	62.7 (2.5) c	725.75	<0.001
Fe (mg.dm^-3^)	27.7 (7.9)	22.3 (1.1)	26.0 (0)	0.81	0.470
Mn (mg.dm^-3^)	24.4 (1.5) a	29.8 (0.5) b	41.6 (0.4) c	221.11	<0.001
Zn (mg.dm^-3^)	0.72 (0.04) a	3.77 (1.69) ab	6.77 (5.58) b	5.01	0.030

Mean values, standard deviations (in parentheses) and P values. Differences amongst the experiments were tested by means of ANOVA followed by Tukey test (N = 3). Different letters indicate significant differences in the concentration of the given parameter (P<0.05). Soil methods are described in Section 2.1.

## Discussion

It is indisputable that P inputs boost maize metabolism [46], which is corroborated by the higher biomass and stem diameter of the plants under P fertilization in this study. Under natural non-fertilized conditions, however, maize uses other strategies to overcome P limitation, as indicated by the higher root phosphatase activity in the control in related to P fertilized conditions.

Higher P availability inhibits rPME activity [47] because the plant does not need to produce the enzyme when its product is abundant, which explains the lowest activities in P fertilized plants in both studies using inbred lines. For maize (*Zea mays*), the effect of P addition upon metabolism varies according to the genotype and to its susceptibility to soil P deficiency [48]. The genetic regulation system normally acts to avoid the unnecessary expression of genes in specific organs and in specific time periods. Any deviation from this pattern indicates variation in the regulatory genes system, as observed in hybrid 11, which had higher rPME under P fertilization, similarly to the genotype studied by Wei et al. [20] that detected higher PME under NPK plus maize straw treatment in comparison to unfertilized control plants. Most inbred lines investigated in this study did not respond significantly to P addition, showing that for these experimental genotypes, genes acting on the regulation of rPME are generally not sensitive to soil P availability.

Inbred lines 4, 8 and 12 showed different responses to P fertilization when cultivated in the glasshouse and in the field. While their rPME values were higher in the control than in the fertilized pots in the glasshouse, such differences were not detected in the field. Since higher activities were always detected in the field than in the glasshouse in both treatments, we speculate that the higher soil N availability in the field when compared to the glasshouse may have stimulated the activity of the enzyme since it is N-rich and therefore highly controlled by N [47, 49, 50], especially when soil P contents are low [51]. Another possible explanation for the higher rPME observed in the field resides in the presence of neighbouring plants in the field, whose root contact stimulates rPME, as shown by Lannes et al. [52] for Cerrado wild plants and more recently in a meta-analysis by Chen et al. [53].

When grown alone in the glasshouse pots, the inbred lines presented reduced rPME in relation to the field presumably because they tend to invest more N in plant development and therefore have less N to employ in other strategies, as rPME. In the glasshouse control pots, however, the inbred lines invested more in rPME due to the lower soil P availabilities, at the cost of lower growth, which suggests a tradeoff between high rPME and N economy. These observations point to a low availability of N in the soil as a limiting factor for plant growth when it invests in rPME and does not have sufficient N for growth [49].

The positive effect that rPME exerts on the weight of 100 grains in the P unfertilized hybrids demonstrates, for the first time, the importance of the activity of this enzyme for productivity enhancement in maize under low P conditions and reinforces the need to consider this variable for P acquisition in natural, non-P fertilized agricultural systems. Since it not common that plant breeding companies test their hybrids under natural unfertilized conditions and due to the high number of hybrids currently available in the market, it is possible that some of these genotypes already have high N-use efficiency and high rPME that could make them suitable for more sustainable systems without problems associated to productivity loss.

The positive correlation found between rPME activity and grain filling found in this study partly explains why Wei et al. [20] did not find maize productivity losses or soil phosphatase activity gains after reducing P-NPK fertilization in 20% (similarly to our 12 hybrids that did not respond to P addition). This was possibly because of the stoichiometric regulation of the balance between N and P since it is not convenient for the plant to invest in acid phosphatase activity (and therefore invest nitrogen) under higher phosphate availability. On another hand, to invest in phosphatase activity is desirable to keep a balanced internal N and P plant stoichiometry and thus a better plant development and higher productivity under conditions of low soil phosphate availability, contributing to avoid a fall in productivity and enabling the reduction of the use of phosphate fertilizers.

Lu et al. [54] promoted the superexpression of the genes *OsPAP10a* and *OsPAP10c* in genetically modified rice and Wang et al. [55] inserted the *Arabidopsis thaliana* gene *AtPAP15* in genetically modified soybeans aiming at increasing rPME. Although they observed a better efficiency on organic-P use in soils with low inorganic P availability, the yield was still lower in comparison to the unmodified genotypes. These experiments did not consider N as an important regulation factor for rPME. We reinforce that high availability or use efficiency of N is necessary to translate high rPME to productivity gain, given that this enzyme is highly N demanding [47, 49]. Nitrogen availability and rPME acting to release P seem to colimit grain filling and plant development because as rPME increases, more internal N in used, reducing the availability of N for plant metabolism and thus affecting plant growth [51]. Therefore, we suggest that for optimum productivity and/or growth increase in maize, it is necessary that N availability and rPME increase concomitantly, independently of the soil type. The application of this practice could support a more sustainable maize production system, which could be even more effective if genotypes with N-fixing capacity are used, as in association with diazotrophic bacteria, mainly *Azospirillum brasilense* that can provide from 29% to 82% of the overall N absorbed by maize [56]. Conventional plant breeding allows the indirect selection of genes that are unknown to influence phosphatase, as for example nitrogen-related genes that might be indirectly selected in this process.

The differences in rPME activity found amongst the inbred lines show that genetic variability for this character exists (Table 1), agreeing with results shown by Machado and Furlani [57], which evaluated six genotypes (three common and three improved varieties) and identified one genotype with significantly a lower PME activity in comparison to the others. The occurrence of genetic variability was also detected by Chen et al. [58], who estimated the broad-sense heritability as 52.7% for rPME and as 90.9% for P absorption efficiency in maize. Chen et al. [58] also reported that the two tested parentals differed significantly for rPME, with the P-deficiency tolerant genotype having higher values than the P-deficiency susceptible genotype.

However, no differences were detected in rPME of the commercial hybrids investigated, but the comparison of their rPME under fertilization to the field fertilized inbred lines (1376±478 and 930±330 μmol pNPP g-root^-1^.h^-1^ respectively, F = 13.06, P = 0.001) shows that the indirect selection resulting from genetic improvement tends to select low rPME genotypes, which is corroborated by the high phenotypic and genetic potential of the inbred lines. This makes possible the generation of single-cross with high variability for this character, considering that PME is a quantitative character with 33 genes involved on its control, whose functions are still not well understood [24].

With this work, we show that there is possibility of success on the selection of genotypes of maize that are naturally adapted to low soil P availability, and concomitantly of genotypes that have high rPME under P fertilization to reduce the amount of inorganic P needed to grow maize in the Cerrado. These will help farmers who base their maize production on high technology and will also benefit conventional farmers by reducing the amount of P needed for production. Future research should specifically investigate the relationship between N availability and rPME in maize, by targeting on tests of hybrids without P addition. Considering that the current increases in carbon and N due to human activities are not followed by increases in P availability [59] and that such unbalanced stoichiometries can cause losses for agriculture, we recommend the generation of genetic variability for rPME together with improving the N absorption and use efficiency in maize aiming at productivity increase in more sustainable production systems.

## Conclusions

Previous works on the topic have investigated the relationships between acid phosphatase activity and phosphorus deficiency in maize, showing that phosphatase activity increased under extremely low soil phosphorus availability, but also generating very low yields.

This study found phenotypic variability for root acid phosphatase activity in maize cultivated in Brazil, being important for plant development and cultivation in the diverse Brazilian soils. We also observed a possible modulation of phosphatase activity by nitrogen. Due to its high genetic potential, and for positively influencing grain productivity in low P soils, the insertion of root acid phosphatase activity in programs of maize genetic improvement aiming at a higher P efficiency is promising. The development of genotypes adapted to more sustainable production systems in P impoverished areas in Brazil could reduce P fertilizer use, mitigating eutrophication in Cerrado natural adjacent ecosystems.

## Supporting information

S1 Fig Maize plants growing in the field and in the glasshouse photos. Maize plants growing in the field (a,c) and in the glasshouse (b) for assessment of the effects of genotype on phosphatase activity (d,e) and of this upon productivity (f) in Brazil. Photo credits: a, b, c–Luciola S Lannes; d, e–Lucas Lopes e Silva; f–João Antonio da Costa Andrade.
(DOCX)

S2 Fig Schematic diagram showing the field design of field study 1. The three genotypes that had significantly lower rPME in the control than in P fertilized pots in the Glasshouse study (L4, L8 and L12, S1 Table), and a border flint maize population, selected for low technology, genetically variable and equilibrated were grown under Control (only water added), or NPK fertilization (20 kg.ha^-1^ (N): 51.6 kg.ha^-1^ (PO_4_): 33.2 kg.ha^-1^ (K)). The plants were grown on lines with 3 meters length each with inter-row distance of 0.90 m. This experiment was not randomized due to the aim of performing the cross-pollination manually, to obtain single-cross maize hybrids, where each genotype on its line was parallel with another line of a inbred lines of interest for cross-pollination. With all possible combinations being carried out, the population was planted around the inbred lines and their collection was random within each soil fertilization treatment. Single asterisks indicate where samples were collected for analyses.
(DOCX)

S3 Fig Schematic diagram (left) and identification of the hybrids for each block (right) showing the field design of Field study 2. We used 13 commercial hybrids (S2 Table), in three randomized blocks (red and black numbers in the left scheme were used to delimitate block, 1, 2 and 3). Two treatments were applied: Control–NK addition (20 kg.ha^-1^ (N): 0 (PO_4_): 33.2 kg.ha^-1^ (K)), or NPK fertilization (20 kg.ha^-1^ (N): 51.6 kg.ha^-1^ (PO_4_): 33.2 kg.ha^-1^ (K)). The plants were grown on 6 lines with 5 meters length each with inter-row distance of 0.45 m, in a total density equivalent to 60,000 plants per hectare. Root phosphatase activity was measured in one random plant from each block (total of 78 plants). Number of leaves, root plant water content, plant height and ear height were measured in five random plants from each block (total of 390 plants). Per block 100 grains were randomly selected for dry weight measurement.
(DOCX)

S1 Table Identification of the inbred lines of maize (*Zea mays*) studied in the glasshouse study and in field study 1. *—genotypes used both in the Glasshouse and Field study 1.
(DOCX)

S2 Table Identification and characterization of the 13 commercial hybrids of maize (*Zea mays*) used in field study 2. (DOCX)

S3 Table Soil characteristics in the glasshouse study. Mean values, standard deviations (in parentheses) and t tests results for soil characteristics in control and P fertilized pots (200 mg.P.kg^-1^ soil).
(DOCX)

S4 Table Mean values, standard deviations (in parentheses), and P values associated to t tests checking the effect of P fertilization (200 mg.P.kg^-1^ soil) on measured variables in 17 inbred lines in the 9-leaf stage of maize (*Zea mays*) from the glasshouse study. N = 3.
(DOCX)

S5 Table Mean values and standard deviations of plant height (cm) and ear height (cm) of 13 commercial hybrids of maize cultivated in the field study 2 under two treatments. Control–NK addition (20 kg.ha^-1^ (N): 0 (PO_4_): 33.2 kg.ha^-1^ (K), or NPK fertilization (20 kg.ha^-1^ (N): 51.6 kg.ha^-1^ (PO_4_): 33.2 kg.ha^-1^ (K)). The effects of fertilization upon measured variables were tested by means of Student t tests (N = 3) and indicated by asterisks when significant (* P<0.05, ** P<0.01). Differences among hybrids were analysed through ANOVA followed by Tukey test (N = 3) and are indicated by different letters within the treatment (P<0.05).
(DOCX)

S6 Table Values of F, significance and coefficient of determination resulting from single linear regressions between productivity and growth variables and rPME in the commercial hybrids (field study 2). Data are show for control, P fertilized and all data analysed together.
(DOCX)
