# Supplementary figures and images for: Potential of root acid phosphatase activity to reduce phosphorus fertilization in maize cultivated in Brazil
Source: PLoS One. 2023 Oct 27;18(10):e0292542. doi: 10.1371/journal.pone.0292542 (PMC10610443; doi:10.1371/journal.pone.0292542)

S1 Fig.


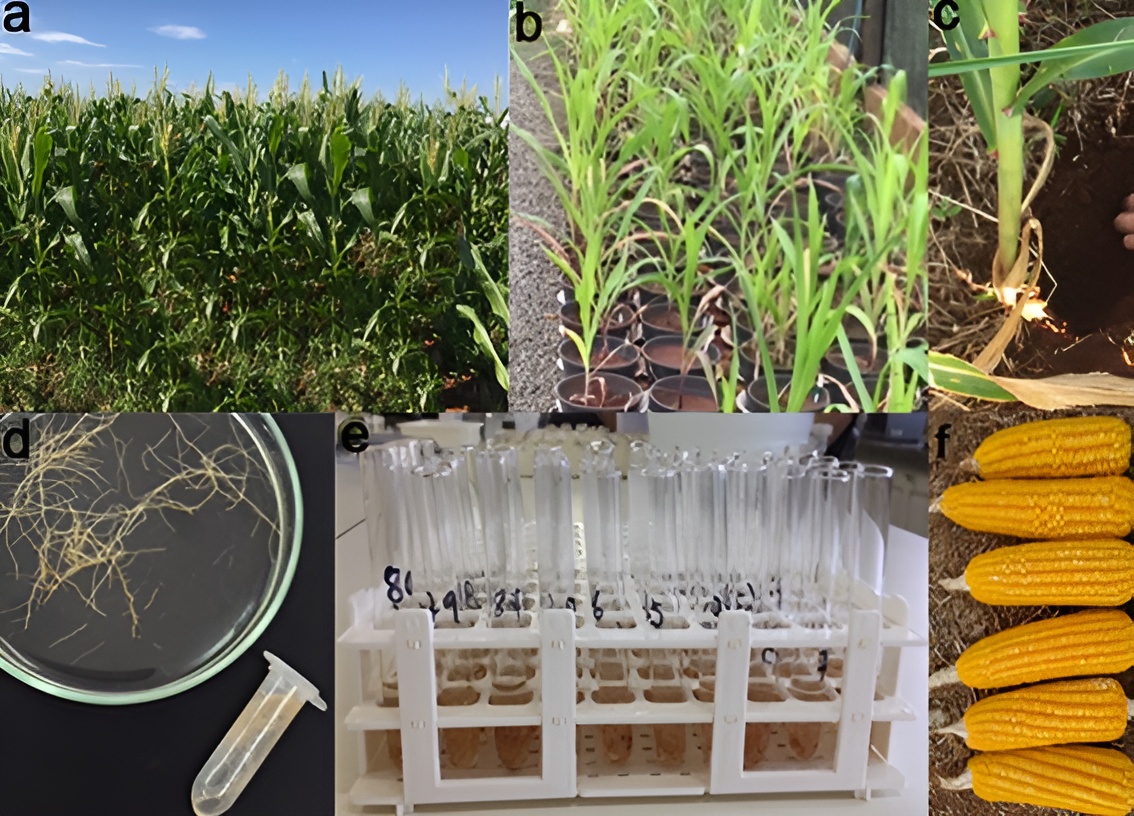

Supplement: S1 Fig — Maize plants growing in the field (a,c) and in the glasshouse (b) for assessment of the effects of genotype on phosphatase activity (d,e) and of this upon productivity (f) in Brazil. Photo credits: a, b, c–Luciola S Lannes; d, e–Lucas Lopes e Silva; f–João Antonio da Costa Andrade. (DOCX) [file pone.0292542.s001.docx]

**S2 Fig.**


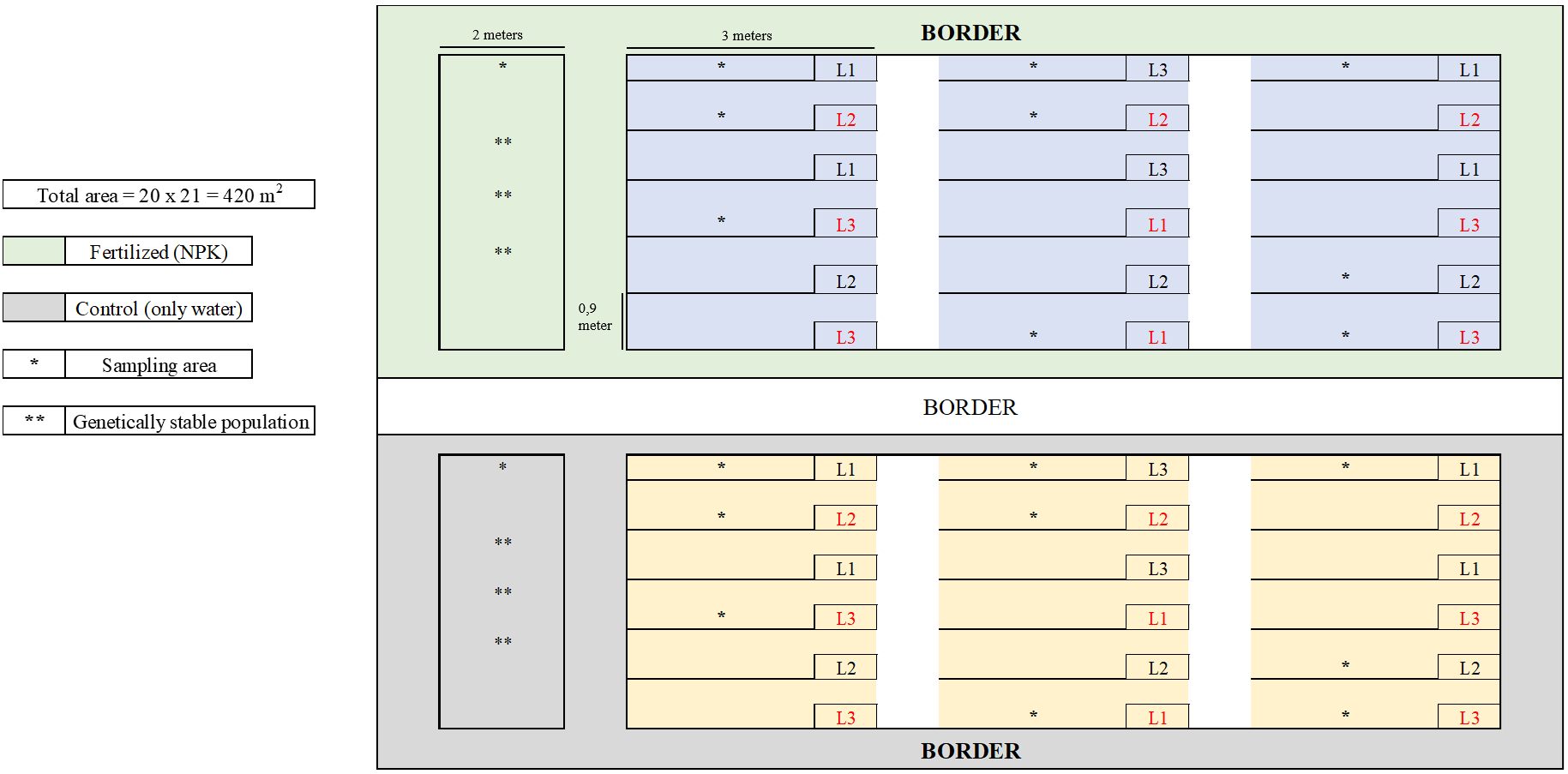

Supplement: S2 Fig — The three genotypes that had significantly lower rPME in the control than in P fertilized pots in the Glasshouse study (L4, L8 and L12, S1 Table), and a border flint maize population, selected for low technology, genetically variable and equilibrated were grown under Control (only water added), or NPK fertilization (20 kg.ha-1 (N): 51.6 kg.ha-1 (PO4): 33.2 kg.ha-1 (K)). The plants were grown on lines with 3 meters length each with inter-row distance of 0.90 m. This experiment was not randomized due to the aim of performing the cross-pollination manually, to obtain single-cross maize hybrids, where each genotype on its line was parallel with another line of a inbred lines of interest for cross-pollination. With all possible combinations being carried out, the population was planted around the inbred lines and their collection was random within each soil fertilization treatment. Single asterisks indicate where samples were collected for analyses. (DOCX) [file pone.0292542.s002.docx]

**S3 Fig.**


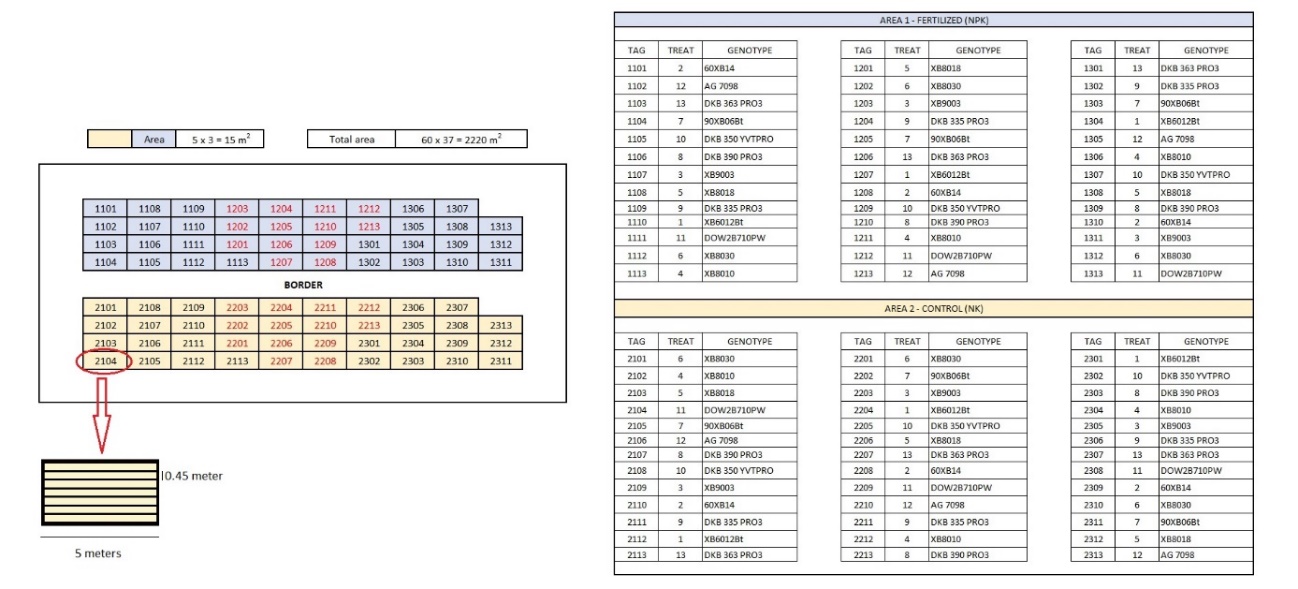

Supplement: S3 Fig — Schematic diagram (left) and identification of the hybrids for each block (right) showing the field design of Field study 2. We used 13 commercial hybrids (S2 Table), in three randomized blocks (red and black numbers in the left scheme were used to delimitate block, 1, 2 and 3). Two treatments were applied: Control–NK addition (20 kg.ha-1 (N): 0 (PO4): 33.2 kg.ha-1 (K)), or NPK fertilization (20 kg.ha-1 (N): 51.6 kg.ha-1 (PO4): 33.2 kg.ha-1 (K)). The plants were grown on 6 lines with 5 meters length each with inter-row distance of 0.45 m, in a total density equivalent to 60,000 plants per hectare. Root phosphatase activity was measured in one random plant from each block (total of 78 plants). Number of leaves, root plant water content, plant height and ear height were measured in five random plants from each block (total of 390 plants). Per block 100 grains were randomly selected for dry weight measurement. (DOCX) [file pone.0292542.s003.docx]
