# Supplementary material for: Potential of root acid phosphatase activity to reduce phosphorus fertilization in maize cultivated in Brazil
Source: PLoS One. 2023 Oct 27;18(10):e0292542. doi: 10.1371/journal.pone.0292542 (PMC10610443; doi:10.1371/journal.pone.0292542)
Supplement: S1 Table — *—genotypes used both in the Glasshouse and Field study 1. (DOCX) [file pone.0292542.s004.docx]

**S1 Table.**

| Code | Inbred lines name | Type of height |
| --- | --- | --- |
| L1 | 9D | Normal |
| L2 | 8F | Brachitic |
| L3 | IVF1-5 | Brachitic |
| L4 | IVD1-3* | Brachitic |
| L5 | 6F | Normal |
| L6 | IVD1-5 | Brachitic |
| L7 | 10F | Normal |
| L8 | IVF1-230-1-3-1-2-1-2-1* | Brachitic |
| L9 | CON-15-2-1-1-1-1-1-1 | Normal |
| L10 | ND1-4-2-1-1-4-1-1 | Normal |
| L11 | IVF1-242-1-1-3-2-2-1 | Brachitic |
| L12 | CON-23-1-1-1-1-1-2-1* | Normal |
| L13 | IVD1-6 | Brachitic |
| L14 | IVF1-249-2-1-1-3-1-5-2 | Brachitic |
| L15 | IVD1-8 | Brachitic |
| L16 | IVF1-52-1-1-4-3-1-1-1 | Brachitic |
| L17 | IVF1-193-1-2-1-1-1-1 | Brachitic |
| Pop | Flint maize population, selected for low technology, genetically variable and equilibrated |  |
