# Supplementary material for: Potential of root acid phosphatase activity to reduce phosphorus fertilization in maize cultivated in Brazil
Source: PLoS One. 2023 Oct 27;18(10):e0292542. doi: 10.1371/journal.pone.0292542 (PMC10610443; doi:10.1371/journal.pone.0292542)
Supplement: S2 Table — (DOCX) [file pone.0292542.s005.docx]

**S2 Table.**

| Code | Identification | Type | Cycle | Transg./Conv. |
| --- | --- | --- | --- | --- |
| H1 | XB 6012 Bt | Single-cross | Early | Transgenic |
| H2 | 60XB14 | Single-cross | Early | Conventional |
| H3 | XB 9003 | Single-cross | Super-early | Conventional |
| H4 | XB 8010 | Double-cross | Super-early | Conventional |
| H5 | XB 8018 | Double-cross | Early | Conventional |
| H6 | XB 8030 | Double-cross | Early | Conventional |
| H7 | 90XB06 Bt | Single-cross | Super-early | Transgenic |
| H8 | DKB 390 PRO3 | Single-cross | Early | Conventional |
| H9 | DKB 335 PRO3 | Single-cross | Early | Conventional |
| H10 | DKB 350 YVTPRO | Single-cross | Early | Conventional |
| H11 | DOW 2B710 PW | Single-cross | Super-early | Conventional |
| H12 | AG 7098 | Single-cross | Semi-early | Conventional |
| H13 | DKB 363 PRO3 | Single-cross | Early | Conventional |
