# Supplementary material for: Potential of root acid phosphatase activity to reduce phosphorus fertilization in maize cultivated in Brazil
Source: PLoS One. 2023 Oct 27;18(10):e0292542. doi: 10.1371/journal.pone.0292542 (PMC10610443; doi:10.1371/journal.pone.0292542)
Supplement: S4 Table — N = 3. (DOCX) [file pone.0292542.s007.docx]

**S4 Table.**

| Variables | Control | P fertilized | P |
| --- | --- | --- | --- |
| rPME (μmol pNPP g-root^-1^ h^-1^) | 610 (224) | 505 (207) | 0.003 |
| Height (cm) | 64 (11) | 61 (16) | 0.170 |
| Number of leaves (unit) | 9.5 (1.2) | 10.2 (1.8) | 0.055 |
| Stem diameter (cm) | 8.5 (2.2) | 9.8 (1.8) | <0.001 |
| Root biomass (g) | 1.46 (0.78) | 1.74 (1.28) | 0.844 |
| Aerial biomass (g) | 2.56 (1.04) | 3.24 (1.65) | 0.039 |
| Total biomass (g) | 4.03 (1.60) | 4.99 (2.71) | 0.169 |
| Root water content (%) | 88.31 (2.46) | 89.50 (1.52) | 0.004 |
