# Supplementary material for: Potential of root acid phosphatase activity to reduce phosphorus fertilization in maize cultivated in Brazil
Source: PLoS One. 2023 Oct 27;18(10):e0292542. doi: 10.1371/journal.pone.0292542 (PMC10610443; doi:10.1371/journal.pone.0292542)
Supplement: S5 Table — Control–NK addition (20 kg.ha-1 (N): 0 (PO4): 33.2 kg.ha-1 (K), or NPK fertilization (20 kg.ha-1 (N): 51.6 kg.ha-1 (PO4): 33.2 kg.ha-1 (K)). The effects of fertilization upon measured variables were tested by means of Student t tests (N = 3) and indicated by asterisks when significant (* P<0.05, ** P<0.01). Differences among hybrids were analysed through ANOVA followed by Tukey test (N = 3) and are indicated by different letters within the treatment (P<0.05). (DOCX) [file pone.0292542.s008.docx]

**S5 Table.**

| Hybrid | Plant height | |  | Ear height | |
| --- | --- | --- | --- | --- | --- |
|  | Control | P fertilized |  | Control | P fertilized |
| H1 | 222 (5.3)** bcde | 200 (2.0) |  | 127 (12.2) cd | 121 (5.0) b |
| H2 | 245 (4.7)** e | 212 (23.5) |  | 130 (11.2)* d | 114 (20.0) ab |
| H3 | 215 (2.0)* abcd | 202 (8.7) |  | 96 (5.8) a | 103 (3.8) ab |
| H4 | 195 (14.5) a | 197 (15.1) |  | 95 (9.8) a | 106 (15.5)* ab |
| H5 | 227 (9.8) bcde | 217 (14.5) |  | 119 (12.7) bcd | 119 (4.5) b |
| H6 | 214 (18.8) abc | 205 (6.0) |  | 112 (25.2) abcd | 118 (10.8) b |
| H7 | 206 (16.5) ab | 202 (9.4) |  | 96 (14.4) a | 99 (3.5) a |
| H8 | 214 (6.4)** abc | 196 (8.7) |  | 124 (1.0) cd | 120 (13.6) b |
| H9 | 231 (7.5)** bcde | 200 (7.0) |  | 106 (20.5) abc | 121 (8.3) b |
| H10 | 210 (15.0) abc | 201 (8.5) |  | 96 (10.4) a | 105 (8.5)*ab |
| H11 | 210 (19.9) abc | 193 (27.2) |  | 99 (13.8) ab | 104 (7.0) ab |
| H12 | 240 (10.0)** de | 215 (24.0) |  | 132 (4.2) d | 126 (13.6) b |
| H13 | 232 (16.7) cde | 216 (14.5) |  | 121 (9.5) bcd | 118 (13.2) b |
| Total | 220 (24.7)** | 204 (25.9) |  | 112 (22.1) | 113 (16.8) |
| P and | P<0.001 | P=0.253 |  | P<0.001 | P<0.001 |
| F (ANOVA) | F=6.90 | F=1.25 |  | F=9.87 | F=4.57 |
