# Supplementary material for: Potential of root acid phosphatase activity to reduce phosphorus fertilization in maize cultivated in Brazil
Source: PLoS One. 2023 Oct 27;18(10):e0292542. doi: 10.1371/journal.pone.0292542 (PMC10610443; doi:10.1371/journal.pone.0292542)
Supplement: S6 Table — Data are show for control, P fertilized and all data analysed together. (DOCX) [file pone.0292542.s009.docx]

**S6 Table.**

|  | Weight of  100 grains | Plant height | Ear height | Number of leaves |
| --- | --- | --- | --- | --- |
| *Total* |  |  |  |  |
| F | 1.997 | 0.312 | 1.690 | 0.884 |
| P | 0.162 | 0.578 | 0.198 | 0.350 |
| R^2^ | 0.027 | 0.040 | 0.023 | -0.012 |
| *Control* |  |  |  |  |
| F | 5.394 | 1.127 | 0.268 | 0.824 |
| P | 0.026 | 0.295 | 0.608 | 0.370 |
| R^2^ | 0.130 | 0.030 | 0.007 | -0.022 |
| *Fertilized* |  |  |  |  |
| F | 0.032 | 0.106 | 1.968 | 0.736 |
| P | 0.858 | 0.746 | 0.169 | 0.397 |
| R^2^ | 0.001 | 0.003 | 0.053 | -0.021 |
